# COVID-19-Related Fear and Health-Related Safety Behavior in Oncological Patients

**DOI:** 10.3389/fpsyg.2020.01984

**Published:** 2020-08-05

**Authors:** Venja Musche, Alexander Bäuerle, Jasmin Steinbach, Adam Schweda, Madeleine Hetkamp, Benjamin Weismüller, Hannah Kohler, Mingo Beckmann, Ken Herrmann, Mitra Tewes, Dirk Schadendorf, Eva-Maria Skoda, Martin Teufel

**Affiliations:** ^1^Clinic for Psychosomatic Medicine and Psychotherapy, LVR University Hospital Essen, University of Duisburg-Essen, Essen, Germany; ^2^West German Cancer Center, University Hospital Essen, Essen, Germany; ^3^Department of Nuclear Medicine, University Hospital Essen, Essen, Germany; ^4^Department of Medical Oncology, University Hospital Essen, Essen, Germany; ^5^Department of Dermatology, University Hospital Essen, Essen, Germany

**Keywords:** anxiety, cancer, coping, COVID-19, distress, oncology, SARS-CoV-2

## Abstract

**Objective:**

This study aimed to assess cancer patients’ psychological burden during the COVID-19 pandemic by investigating distress (distress-thermometer), health status (EQ-5D-3L), general anxiety (GAD-7), COVID-19-related fear and associated behavioral changes and comparing these to matched healthy controls, using propensity score matching (PSM).

**Methods:**

During the first days of the COVID-19 pandemic in Germany, March 16 to 30, 2020, 150 actually treated cancer patients and 150 matched healthy controls participated in this study. Participants completed an anonymous online survey assessing health status, distress, general anxiety, COVID-19-related fear and behavioral changes (i.e., adherent safety behavior and dysfunctional safety behavior).

**Results:**

Cancer patients showed no elevated level of distress, *U* = 10,657.5, *p* = 0.428, general anxiety *U* = 10,015.5, *p* = 0.099, or COVID-19-related fear compared to healthy controls, *U* = 10,948, *p* = 0.680. Both groups showed elevated COVID-19-related fear. Cancer patients reported more adherent safety behavior, such as washing hands more often or avoiding public places, *U* = 8,285, *p* < 0.001, *d* = 0.468. They also reported more dysfunctional safety behavior such as buying larger quantities of basic food, compared to healthy controls *U* = 9,599, *p* = 0.029, *d* = 0.256. Adherent safety behavior could be significantly explained by cancer diagnosis, increased COVID-19-related fear and subjective level of information about COVID-19, *R*^2^ = 0.215, *F*(3) = 27.026, *p* < 0.001.

**Conclusion:**

This suggests that cancer patients are more likely to utilize adherent safety behavior. Cancer patients reported comparable levels of distress and anxiety compared to healthy controls. Still, the COVID-19 pandemic is associated with elevated COVID-19-related fear. Therefore, specific interventions are needed to prevent anxiety and improve mental health during the COVID-19 pandemic.

## Introduction

Since December 2019, the novel coronavirus severe acute respiratory syndrome CoV-2 (SARS-CoV-2), which was first reported in Wuhan City in China, is spreading rapidly throughout the world and places an immense threat and severe challenges on global public health (Zhu et al., 2020). On March 11, 2020, the World Health Organization (WHO) officially classified the spread of SARS-CoV-2 as a pandemic (World Health Organization, 2020b). The number of confirmed cases worldwide has reached 4,248,389, including 292,046 deaths, until May 14th 2020 (World Health Organization, 2020a). The clinical picture of reported SARS-CoV-2 infections seems to be heterogenic, ranging from asymptomatic infections and mild respiratory diseases, to severe viral pneumonia with respiratory failure and even death in a small proportion of patients (Zhou et al., 2020). First studies, investigating possible risk factors for a severe course of disease or worse prognosis and implications of comorbid conditions, emerge. The results of a meta-analysis by Yang et al. (2020) including 46,248 infected patients in China, suggest that the most prevalent comorbid conditions were hypertension (17%) followed by diabetes (8%), cardiovascular disease (5%), and respiratory illness (2%). Overall, patients with a severe course of disease were older and had a higher number of comorbidities than those with a non-severe course of disease (Wang et al., 2020) suggesting older age and comorbid conditions as risk factors for infection and worse prognosis (Yang et al., 2020).

Cancer is one of the most common diseases in the world and an increasingly global health issue (Fitzmaurice et al., 2019). In regard to SARS-CoV-2, only preliminary research of the effect of a comorbid cancer diagnose exists. The prospective investigation by Liang et al. (2020) included 1,590 cases with laboratory-confirmed COVID-19 acute respiratory disease from 575 hospitals. This nationwide analysis revealed a higher percentage of cancer in patients with SARS-CoV-2 than in the general Chinese population (based on the report of cancer epidemiology in China from 2015), suspecting cancer patients being at higher risk of SARS-CoV-2 infection. Due to the very small sample size (*N* = 18) with an average age of 63.1 years (*SD* = 12.1), the results need to be interpreted with caution. In addition, the retrospective study by Zhang et al. (2020) including 28 COVID-19-infected cancer patients from three hospitals in Wuhan, China, with a median age of 65.0 years, revealed poor outcomes from SARS-CoV-2 infection in cancer patients, supporting the vulnerability of this patient group in the present pandemic. Cancer patients are generally more susceptible to infections due to the malignancy itself and its treatment with immunosuppressive agents, chemotherapy, or surgery (Rolston, 2017).

Cancer patients are also a vulnerable patient group due to the psychological burden of their chronic disease (Glaser et al., 1987). In fact, half of the patients with cancer suffer from acute psychological distress and one third meet the criteria for at least one mental health disorder, including anxiety and mood disorders, at a four-week prevalence (Mehnert et al., 2014, 2018). The pandemic might have an impact on mental health due to the resulting uncertainty, public restrictions, physical distancing and effects on our everyday life. Previous research revealed an increased psychological burden during this pandemic, including distress, anxiety, and depression (Rajkumar, 2020). Brooks et al. (2020) examined the psychological effect of quarantine during the pandemic indicating psychological burden for those being unable or not allowed to participate in social life. Further, people with mental disorders might be especially threatened. Yao et al. (2020) suggested an increased vulnerability for worsened mental health conditions in already affected individuals during SARS-CoV-2 pandemic. Juanjuan et al. (2020) investigated 658 breast cancer patients and survivors during the SARS-CoV-2 outbreak in Hubei Province, China. The results revealed a psychological burden including high rates of anxiety, distress, depression, and insomnia in this patient group. The commentary by Nekhlyudov et al. (2020) stressed possible implications of SARS-CoV-2 on physical and psychosocial well-being in cancer survivors and the need for tailored, patient-centered care. Thus, the current pandemic might have not only an effect on physical but also on mental health, especially in case of preexisting vulnerability.

The psychological impact of the pandemic is associated with behavioral changes. A cross-sectional study by Harper et al. (2020) investigated 324 individuals participating in an online survey. The results revealed that fear of the pandemic predicts public health-compliant behaviors, including improved hand hygiene and social distancing, and might be a functional response in the context of the pandemic. Besides the psychological impact of the pandemic affecting behavioral changes, knowledge about COVID-19 and associated protection methods has shown to influence behavioral changes associated with the pandemic. An increased situational awareness (i.e., perceived understanding) has shown to significantly increase the adoption of health behavior (Qazi et al., 2020). Additionally, lower knowledge about COVID-19 has shown to be associated with dysfunctional behavior such as stockpiling (Clements, 2020).

Despite the rapidly growing body of literature on SARS-CoV-2, not only considering somatic but also considering psychological aspects, little is known about the pandemic’s impact on mental health and health related safety behavior of vulnerable patient groups as patients with cancer are. To close this gap, we conducted the present investigation of the psychological burden of the SARS-CoV-2 pandemic on cancer patients. By investigating distress, health status, general anxiety, COVID-19-related fear and associated behavioral changes [i.e., adherent safety behavior (ASB) and dysfunctional safety behavior (DSB)] in cancer patients and comparing these to a propensity matched (10 variables) healthy control group, we aimed to give insights to the psychological burden and the needs of patients with cancer during this pandemic. More specifically, we expected cancer patients, as a vulnerable patient group, to be especially burdened by the current pandemic with increased distress, general anxiety, and COVID-19-related fear compared to healthy controls. Further, cancer patients were expected to report more associated behavioral changes compared to healthy controls. As the literature revealed COVID-19-related behavioral changes to be associated with fear and knowledge about the pandemic, we included these as possible predictors. More specifically, we expect safety behavior, adherent and DSB, to be predicted by cancer diagnosis, COVID-19-related fear, and subjective level of information.

## Materials and Methods

### Procedure

Over the course of 14 days (March 16 to 30, 2020), during the first days of the SARS-CoV-2 pandemic in Germany, we distributed the survey to cancer patients of the University Hospital Essen. They were contacted for follow-up assessment and asked to participate in the anonymous online survey (response rate: 85.79%). The healthy control group was derived out of a German population based community sample (*N* = 12,863) reported in previous research (Bäuerle et al., 2020c; Teufel et al., 2020). The respective anonymous online survey was distributed through online channels. To control for time inconsistency, only participants who answered the survey in the same period (March 16 to 31, 2020) were considered. Eligibility requirement was adult age (≥18 years) for all participants. All participants gave written informed consent. This study was registered at the German Clinical Trials Register with an ethical approval (19-8834-BO) by the medical faculty of the University Duisburg-Essen, registration ID: DRKS00021164.

### Propensity Score Matching

To account for the differences in the two samples, healthy controls were matched to cancer patients using propensity score matching (PSM; Lee and Little, 2017) for conditioning our large set of covariates (Rosenbaum and Rubin, 1983; Thoemmes, 2012). Besides sociodemographic variables, we included mental disorder and somatic diseases as covariates. More specifically, cardiovascular disease, respiratory disease, hypertension, and diabetes are associated with an increased psychopathology (Mikkelsen et al., 2004; Collins et al., 2009; Cohen et al., 2015; Pan et al., 2015). Further, as individuals suffering from these diseases are considered especially vulnerable to SARS-CoV-2 (Yang et al., 2020) they might feel even more burdened. Therefore, these variables were included as covariates. Cases were matched using the following covariates with percent balance improvement included: education (100.00%), age (98.89%), marital status (96.38%), gender (86.49%), city size (75.58%), somatic diseases including hypertension (94.60%), cardiovascular diseases (91.91%), diabetes (64.26%), and respiratory diseases (53.47%), as well as mental disorders (91.96%). To estimate the effect of a specific variable, in our case cancer diagnosis, PSM attempts to remove bias due to the observed confounding covariates (Rosenbaum and Rubin, 1983). Inferences about cancer diagnosis made by using PSM are valid only if both groups have similar distributions of baseline covariates (Austin, 2009). The propensity score was estimated using logistic regression. We checked that the propensity score and covariates were balanced across both groups. Each cancer patient was matched to a healthy control on propensity score. Further analysis was based on this new sample. Further details can be found in Supplementary Table S1.

### Assessment Instruments

Socio-demographic data was assessed including age, gender, education, marital status, occupation, residential situation, as well as physical and psychological health status. Additionally, in cancer patients type of cancer, tumor-stage, and current treatment (e.g., adjuvant treatment and palliative treatment) was assessed. All data was self-reported.

Health status was assessed using the visual analog scale item from the European Quality of Life 5 Dimensions 3 Level Version (EQ-5D-3L). The scale ranges from “0 = the worst health you can imagine” to “100 = the best health you can imagine” (EuroQol Group, 1990).

Self-reported anxiety and its severity was assessed by use of the Generalized-Anxiety-Disorder (GAD-7) questionnaire on a 4-point scale, ranging from “0 = not at all” to “3 = nearly every day.” Scores of 5, 10, and 15 are taken as cut-off points for mild, moderate and severe anxiety, respectively (Spitzer et al., 2006). The internal consistency was high with a Cronbach’s α of 0.901.

For the measurement of distress, we used the German Version of the distress-thermometer. It is a visual analog scale ranging from “0 = no distress” to “10 = extreme distress” (Mehnert et al., 2006).

For the assessment of subjective level of information about COVID-19 and recommended protection measures (e.g., “I feel informed about COVID-19”), three items were formulated. Answers were given on a 7-point Likert-scale ranging from “1 = strongly disagree” to “7 = strongly agree.” The internal consistency was high with a Cronbach’s α of 0.804.

COVID-19-related fear was assessed by one item (“I worry about COVID-19”). Answers were given on a 7-point Likert-scale ranging from “1 = strongly disagree” to “7 = strongly agree.” Thus, higher scores indicate higher COVID-19-related fear.

For the measure of ASB and DSB, nine items were formulated to cover general recommendations by the World Health Organization (2020b) including physical distancing and increased hand hygiene, and reported behavioral changes in media including stockpiling. Before further analysis, the reliability was tested and one item had to be excluded. Based on the rotated component analysis by Varimax the two sub-scales were identified: the 4-item sub-scale of ASB (*M* = 6.19, *SD* = 1.03) with Cronbach’s alpha of 0.738, and the 4-item sub-scale of DSB (*M* = 2.92, *SD* = 1.24) with Cronbach’s alpha of 0.770. Answers were given on a 7-point Likert-scale ranging from “1 = strongly disagree” to “7 = strongly agree.” The items of the two sub-scales and the corrected item-scale correlations can be found in Supplementary Table S2.

### Statistical Analyses

Statistical analyses were carried out by use of *statistical program for social sciences SPSS* version 26 (IBM, New York). The level of significance for all analyses was set at α = 0.05. By construction there was no missing data as the survey could only be completed when all items were answered. The chi-square tests confirmed no differences in sociodemographic data between the two groups (Table 1). The Kolmogorov-Smirnov normality test indicated a non-normal distribution. Thus, we relied on non-parametric tests. For correlations, Spearman’s *rho* was calculated. The VIF values showed no indication of multicollinearity between the predictors (Hair et al., 1995). To investigate the differences between cancer patients and healthy controls in health status, general anxiety, COVID-19-related fear, distress, subjective level of information, and associated behavioral changes, nonparametric Mann-Whitney-*U*-tests were used. Further, to test whether cancer diagnosis (dummy coded with 0 = no and 1 = yes), COVID-19-related fear and subjective level of information do predict ASB and DSB, we constructed two multiple regression models.

**TABLE 1:** Sociodemographic and medical characteristics.

	**Cancer**		**Healthy**		
	**patients**		**controls**		
	**N**	**%**	**N**	**%**	
**Sex**					χ^2^ (1) = 0.213, *p* = 0.644
Female	78	52.0	74	49.3	
Male	72	48.0	76	50.7	
**Age**					χ^2^ (2) = 0.954, *p* = 0.621
<45 years	17	11.3	18	12.0	
45–75 years	122	81.4	125	83.3	
>75 years	11	7.3	7	4.7	
**Marital status**					χ^2^ (4) = 7.492, *p* = 0.112
Single	13	8.7	23	15.3	
Married	110	73.3	93	62.0	
In a relationship	15	10.0	15	10.0	
Divorced/separated	6	4.0	14	9.3	
Widowed	6	4.0	5	3.3	
**Educational level**					χ^2^ (4) = 5.466, *p* = 0.243
University education	50	33.3	56	37.3	
Higher education entrance qualification	46	30.7	32	21.3	
Intermediate secondary education	38	25.3	37	24.7	
Lower secondary education	16	10.7	24	16.0	
No qualification	0	0	1	0.7	
**Employment**					
Full employment	36	24.0	−	−	
Partial employment	15	10.0	−	−	
Not employed	73	48.7	−	−	
Sick leave	25	16.7	−	−	
Other	1	0.7	−	−	
**City size**					χ^2^ (3) = 2.726, *p* = 0.436
100,000 residents	82	54.7	86	57.3	
20,000 residents	43	28.7	33	22.0	
5,000 residents	16	10.7	23	15.3	
<5,000 residents	9	6.0	8	5.3	
**Tumor Stage**					
I	10	6.7	−	−	
II	11	7.3	−	−	
III	21	14.0	−	−	
IV	36	24.0	−	−	
Unknown	72	48.0	−	−	
**Treatment situation**					
Curative	24	16.0	−	−	
Palliative	24	16.0	−	−	
Cured	37	24.7	−	−	
Could not be assessed	30	20.0	−	−	
Currently not decidable	35	23.3	−	−	
**Type of cancer**					
Bone cancer, cartilage tumor, soft-tissue sarcoma	11	7.3	−	−	
Breast cancer	8	5.3	−	−	
Cancer of the central nervous system	7	4.7	−	−	
Cancer of the gastrointestinal tract	23	15.3	−	−	
Cancer of the eye	6	4.0	−	−	
Head and neck cancer	10	6.7	−	−	
Leukemia or Lymphoma	7	4.7	−	−	
Lung cancer	17	11.3	−	−	
Skin cancer	36	24.0	−	−	
Thyroid cancer	12	8.0	−	−	
Urogenital cancer	13	8.7	−	−	
Total	150	100	150	100	

*Cancer of the gastrointestinal tract: anal, colorectal, esophageal, gallbladder, gastric, kidney, liver, and pancreatic cancers. Cancer of the eye: retina cell carcinoma, retinoblastoma and uveal melanoma. Urogenital cancer: bladder, cervical, and prostate cancers.*

## Results

### Participant Characteristics

The participants were 150 cancer patients and 150 healthy controls, who were matched by ten variables to the cancer patients based on their demographic data. Most of the cancer patients (52% female) were older than 45 years (88.7%) and married (73.3%). One third had completed a university degree (31.9%) and most of the participants were not employed due to, e.g., retirement (66%). The patients suffered from different types of cancer in various tumor-stages, with a mean of 30.85 months (*SD* = 45.695) since diagnosis (including first diagnosis, recidivism, and metastatic cancer) and 44.7% reporting metastatic cancer. The current treatment differed from palliative (16%), over adjuvant (16%) to adjuvant treatment completed (24.7%). The 150 healthy controls were matched as mentioned above: 49.3% female, mostly aged between 45 and 74 years (83.3%), married (62%) and living in cities with populations with more than 20,000 residents (79.3%). A total of 37.3% of the healthy controls had a completed university degree. For sample descriptions, see Table 1.

### Comparison Between Cancer Patients and Healthy Controls

The results of the Mann-Whitney-*U*-tests showed no significant differences between cancer patients and healthy controls in regard to distress, *U* = 10,657.5, *p* = 0.428, general anxiety, *U* = 10,015.5, *p* = 0.099, and COVID-19-related fear, *U* = 10,948, *p* = 0.680. Cancer patients (*M* = 5.17, *SD* = 1.582), and healthy controls (*M* = 5.06, *SD* = 1.676) reported elevated COVID-19-related fear. Both groups reported to be equally well informed about COVID-19, *U* = 11,220, *p* = 0.967. In comparison to healthy controls, cancer patients showed a lower health status, *U* = 6,486, *p* < 0.001, *d* = 0.787. In regard to safety behavior, cancer patients reported higher levels of ASB, *U* = 8,285, *p* < 0.001, *d* = 0.468, and DSB compared to healthy controls, *U* = 9,599, *p* = 0.029, *d* = 0.256. For an overview see Table 2.

**TABLE 2:** Comparisons between cancer patients and healthy controls.

	**Cancer patients**	**Healthy controls**		
	**(*n* = 150)**	**(*n* = 150)**		
	***M* (*SD*)**	***M* (*SD*)**	***U***	***p***
Distress	4.86 (2.742)	5.13 (3.1529)	10,657.5	0.428
Health status	66.05 (19.257)	78.99 (18.798)	6,486	< 0.001**
General anxiety	5.29 (4.062)	4.95 (4.932)	10,015.5	0.099
COVID-19-related fear	5.17 (1.582)	5.06 (1.676)	10,948	0.680
Subjective level of information	6.147 (0.781)	6.116 (0.842)	11,220	0.967
ASB	6.40 (0.867)	5.97 (1.133)	8,285	< 0.001**
DSB	3.07 (1.179)	2.77 (1.280)	9,599	0.028*

Mean parameter values for each of the analyses are shown for cancer patients (*n* = 150) and healthy controls (*n* = 150), as well as the results of Mann–Whitney *U* tests (assuming unequal variance). ASB, adherent safety behavior; DSB, dysfunctional safety behavior. ** significant at the 0.01 level and * significant at the 0.05 level.

### Prediction of Adherent and Dysfunctional Safety Behaviors

The results of the regression models with cancer diagnosis (dummy coded with 0 = no and 1 = yes), subjective level of information and COVID-19-related fear predicting ASB and DSB are shown in Table 3. Significant predictors of ASB in the whole sample were cancer, subjective level of information and COVID-19-related fear. This model provided an explained variance of 21.5%. DSB was statistically significantly predicted by cancer, COVID-19-related fear and low subjective level of information. However, the explained variance was only 6.9%.

**TABLE 3:** Regression coefficients predicting ASB and DSM.

**DV**	**Predictor**	***b*^a^**	***SE*^a^**	***t***	***p***
ASB	(Intercept)	2.914	0.438	6.652	<0.001
	Cancer diagnosis	0.391	0.106	3.696	<0.001
	Subjective level of information	0.332	0.065	5.077	<0.001
	COVID-19-related fear	0.203	0.033	6.239	<0.001
DSB	(Intercept)	3.175	0.574	5.534	<0.001
	Cancer diagnosis	0.287	0.139	2.067	0.040
	Subjective level of information	–0.191	0.086	–2.230	0.026
	COVID-19-related fear	0.151	0.043	3.536	<0.001

*N = 300. ^*a*^Unstandardized regression coefficients. Model 1: Dependent Variable (DV): adherent safety behavior (ASB). Total *R*^2^ = 0.215, *F*(3) = 27.026, *p* < 0.001. Model 2: DV: dysfunctional safety behavior (DSB). Total *R*^2^ = 0.069, *F*(3) = 7.323, p < 0.001.*

## Discussion

The present study investigated the psychological burden of cancer patients during the COVID-19 pandemic. Cancer patients showed no elevated level of distress, general anxiety, or COVID-19-related fear compared to healthy controls. Both groups showed elevated COVID-19-related fear. Cancer patients reported more ASB and DSB, such as buying larger quantities of basic food, compared to healthy controls. Safety behavior could be significantly explained by cancer diagnosis, increased COVID-19-related fear, and subjective level of information about COVID-19.

Patients with a diagnosis of cancer are a vulnerable group due to their reduced health status, increased vulnerability to infections and increased prevalence of mental health conditions such as anxiety or depression. Therefore, cancer patients were expected to be especially threatened by the current pandemic. The present data, showed no elevated level of distress, general anxiety or COVID-19-related fear in cancer patients compared to propensity matched healthy controls (10 variables). In this context, it is important to mention that both, cancer patients and healthy controls, showed elevated COVID-19-related fear and comparable rates of general anxiety, suggesting both groups feel equally threatened by the current pandemic and the associated uncertainty, public restrictions and effects on everyday life. This stresses the importance of specific, situation-based and low-threshold interventions for cancer patients and threatened people in general to reduce psychological burden (Bäuerle et al., 2020a,b).

Subjective level of information was associated with reduced anxiety. Even if cancer patients are a vulnerable patient group with a reduced health status, they reported no elevated distress or anxiety compared to healthy controls. Previous research stressed the importance of information needs in cancer patients and associated psychological burden. Subjective level of information might be associated with reduced uncertainty and psychological burden and elevated feelings of control and self-efficacy (Faller et al., 2016; Keinki et al., 2016; Parker et al., 2016). Here, the cancer patients reported high level of information about the pandemic and associated behavioral changes to prevent infection. This might be one explanation, why they reported no elevated level of anxiety or distress compared to healthy controls. These findings stress the importance of information about COVID-19 and necessary behavioral changes provided by health professionals and society in general to prevent psychological distress and anxiety. Alternatively, the comparable level of distress, general anxiety and COVID-19-related fear for both groups might be due to psychological impact of the acute threat of the pandemic (Bäuerle et al., 2020c; Casagrande et al., 2020) which might surpass the underlying psychological burden of the cancer diagnosis. As the impact of the COVID-19 pandemic on mental health has shown to vary over time (Teufel et al., 2020) it might be possible that people adapt to the continuing threat of the pandemic. In a longitudinal study, Wang et al. (2020) revealed that the pandemic has a continuing impact on mental health including increased distress, anxiety and depressive symptoms four weeks after the initial outbreak in the general population. Still, the continuing impact of the pandemic on mental health needs to be investigated.

Cancer patients developed adaptive coping strategies to master stressful situations effectively (Livneh, 2000). Here, they reported more ASB, such as washing or disinfecting hands more often or avoiding public places. On the other hand, they also showed more DSB such as buying larger quantities of basic food or toilet paper, in comparison to healthy controls. In contrast to our expectations, the mean differences were only marginal. This could be due to the comparable psychological burden the two groups reported, including increased COVID-19-related fear in both groups. ASB could be significantly predicted by cancer, increased COVID-19-related fear and subjective level of information about COVID-19. Again, this stresses the importance of the subjective level of information about COVID-19 to enable ASB. In accordance with previous research by Harper et al. (2020), COVID-19-related fear has been revealed as predictor of behavior change (e.g., social distancing and improved hand hygiene). In general, cancer diagnosis is associated with fear and uncertainty about the future, clinical levels of distress, anxiety, and depression. However, most patients reestablish average mood and functioning within the year after medical treatment (Andersen et al., 1989). Furthermore, threatening life events such as cancer diagnosis can also be associated with positive changes and an increased functioning, termed as post-traumatic growth (PTG) (Tedeschi and Calhoun, 2004). There is evidence that PTG is very common in cancer survivors since cancer patients already mastered threatening situations in the past due to their diagnosis and treatment (Sharp et al., 2018). Thus, it might be possible that the cancer patients might have reestablished mental health comparable to healthy controls due to PTG or time since diagnosis. Therefore, they are threatened by the pandemic comparable to healthy controls. A meta-analysis concluded that positive coping styles are associated with PTG (Shand et al., 2015). Thus, the reported safety behavior by cancer patients could be interpreted as one possible way of coping with the pandemic.

While cancer diagnosis, COVID-19-related fear, and subjective level of information about COVID-19 predicted ASB by 21%. These variables explained only 6% of the variance in DSB. Previous research, investigating possible predictors of stockpiling, revealed that a perceived threat of COVID-19 predicts toilet paper stockpiling (Garbe et al., 2020). In this study, only up to 12% of variance in stockpiling could be explained by the included predictors. This suggests that further research is needed to identify possible predictors of DSB.

### Study Limitations

The present study investigates the psychological burden of cancer patients during the early phase of the COVID-19 pandemic compared to matched healthy controls based on PSM. PSM is a valuable method for conditioning a large set of covariates. However, there are limitations to consider. As the present study is a cross-sectional investigation, causality cannot be assumed. The responding oncological patient group is heterogenic. However, a substantial proportion of the present sample reported to have completed adjuvant treatment limiting generalizability of the results. Further, due to the anonymous approach non-responders could not be assessed. Thus, possible selection biases and differences between responders and non-responders could not be ruled out. Social desirability has also to be mentioned but as the survey was anonymous, the impact of social desirability could be expected to be limited. Further, as COVID-19-related fear was measured by one item, validity of the assessment could be questioned. The investigation took place in the early phase of the COVID-19 pandemic. As the impact of the COVID-19 pandemic on mental health has shown to vary over time (Teufel et al., 2020) the continuing impact of the pandemic on mental health in cancer patients needs to be investigated.

### Clinical Implications

General anxiety and COVID-19-related fear were negatively associated with subjective level of information. Further, cancer patients reported more ASB, such as washing or disinfecting hands more often or avoiding public places and this was significantly explained by subjective level of information. Thus, the findings of the present study stress the importance of information about COVID-19 and related behavioral changes by health professionals and society in general for ASB and reduced anxiety. Cancer patients showed equal levels of distress, general anxiety and COVID-19-related fear in comparison to a matched healthy control group. However, they reported elevated COVID-19-related fear, stressing the need of specific, situation-based and low-threshold interventions for burdened people and people at risk to prevent increasing psychological burden.

## Conclusion

In conclusion, the COVID-19 pandemic is a threatening situation as it has a substantial impact on our everyday life, including social distancing, quarantine, and uncertainty. Cancer patients show comparable levels of distress and anxiety compared to healthy controls. Still, the COVID-19 pandemic is associated with elevated COVID-19-related fear for both groups. Therefore, specific interventions – also community-based – are needed to prevent anxiety and improve mental health during the COVID-19 pandemic.

## Data Availability Statement

The raw data supporting the conclusions of this article will be made available by the authors, on request.

## Ethics Statement

The studies involving human participants were reviewed and approved by the ethics committee of the medical faculty of the University Duisburg-Essen, Essen, Germany. The patients/participants provided their written informed consent to participate in this study.

## Author Contributions

VM and AB made substantial contributions to the study’s design, actively participated in acquisition of data, statistical analysis, and interpretation of data, and prepared the manuscript. JS and AS actively participated in acquisition of data, statistical analysis, and interpretation of data. MH, BW, and HK actively participated in the interpretation of data and edited the manuscript. MB, KH, MT, and DS made substantial contributions to the study’s conception and revised the manuscript critically for important intellectual content. MT and E-MS made substantial contributions to the study’s conception and design, actively participated in the interpretation of data, and revised the manuscript critically for important intellectual content. All authors contributed to the article and approved the submitted version.

## Conflict of Interest

The authors declare that the research was conducted in the absence of any commercial or financial relationships that could be construed as a potential conflict of interest.
