# Supplementary material for: COVID-19-Related Fear and Health-Related Safety Behavior in Oncological Patients
Source: Front Psychol. 2020 Aug 5;11:1984. doi: 10.3389/fpsyg.2020.01984 (PMC7438892; doi:10.3389/fpsyg.2020.01984)
Supplement: Supplementary file 1 [file Table_1.DOCX]

| **Table S1.** Treatment coded regression coefficients of matching variables on group membership. | | | |
| --- | --- | --- | --- |
| Predictors | Odds Ratios | CI | *p* |
| (Intercept) | 0 | 0.00 – 0.00 | <0.001 |
| Gender | 1.56 | 1.10 – 2.22 | 0.012 |
| Age | 2.16 | 1.88 – 2.51 | <0.001 |
| Education | 1.34 | 1.14 – 1.57 | <0.001 |
| Area | 0.93 | 0.77 – 1.11 | 0.437 |
| Presence of disease | 1.13 | 0.68 – 1.81 | 0.619 |
| Presence of dardiovascular disease | 1.21 | 0.65 – 2.13 | 0.524 |
| Presence of pulmonal disease | 0.85 | 0.48 – 1.42 | 0.558 |
| Hypertension | 1.38 | 0.93 – 2.01 | 0.104 |
| Diabetes | 1.27 | 0.68 – 2.25 | 0.431 |
| Psychiatric disease | 0.86 | 0.47 – 1.46 | 0.589 |
| Marital status | 0.92 | 0.77 – 1.09 | 0.363 |
| Observations | 5364 | | |
| R^2^ Tjur | 0.069 |  |  |
|  |  |  |  |

**Table S2.** Corrected Item-Scale Correlations of adherent and dysfunctional safety behavior.

| Adherent safety behavior (ASB) | | Dysfunctional safety behavior (DSB) | |
| --- | --- | --- | --- |
| Item | Corrected Item-Scale Correlation | Item | Corrected Item-Scale Correlation |
| I wash/disinfect my hands more often. | .292 | I have bought larger quantities of basic food (flour, sugar, noodles, rice, and canned food) or will buy more in the near future. | .682 |
| I increasingly avoid public places/ events. | .656 | I have bought larger quantities of hand disinfection/soap/similar or will buy more in the near future. | .603 |
| I increasingly avoid public transit (subway, tram, bus, train). | .660 | I have bought larger quantities of toilet/hygiene articles or will buy more in the near future. | .679 |
| I have changed my trip/ vacation plans or would change them if I had planned a vacation/trip. | .537 | I have become more selfish in my behavior. | .350 |

*Note*. N = 300. ASB = adherent safety behavior, DSB = dysfunctional safety behavior. Answers were given on a 7-point Likert-scale ranging from “1 = strongly disagree” to “7 = strongly agree”.

**COVID-19-specific items**

**Subjective level of information about COVID-19 and recommended protection methods**

I feel informed about COVID-19.

I feel informed about measures to avoid an infection with COVID-19.

I understand the public health authorities‘ advices regarding COVID-19.

**COVID-19-related fear**

I worry about COVID-19.

**Adherent safety behavior (ASB)**

I wash/disinfect my hands more often.

I increasingly avoid public places/ events.

I increasingly avoid public transit (subway, tram, bus, train).

I have changed my trip/ vacation plans or would change them if I had planned a vacation/trip.

**Dysfunctional safety behavior (DSB)**

I have bought larger quantities of basic food (flour, sugar, noodles, rice, and canned food) or will buy more in the near future.

I have bought larger quantities of hand disinfection/soap/similar or will buy more in the near future.

I have bought larger quantities of toilet/hygiene articles or will buy more in the near future.

I have become more selfish in my behavior.

**Table S3.** Correlation coefficients for study variables.

| Variables | 1. | 2. | 3. | 4. | 5. |
| --- | --- | --- | --- | --- | --- |
| 1. Covid-19-related fear | - |  |  |  |  |
| 1. general anxiety | .336** | - |  |  |  |
| 1. Subjective level of information | -.023 | -.255** | - |  |  |
| 1. ASB | .363** | .207** | .246** | - |  |
| 1. DSB | .161** | .192** | -.152** | 0.213** | - |

*Note.* *N* = 300. ASB = adherent safety behavior, DSB = dysfunctional safety behavior. Spearman’s *rho*. ** Correlation is significant at the 0.01 level (two-tailed).

For both groups, COVID-19-related fear was positively correlated with ASB (*rho* = .363, *p* < .001) and DSB (*rho* = .161, *p* = .005). Subjective level of information about COVID-19 was negatively correlated with general anxiety (*rho* = -.255, *p* < .001), positively correlated with ASB (*rho* = .246, *p* < .001), and negatively correlated with DSB (*rho* = -.152, *p* = .008. COVID-19-related fear and general anxiety were positively correlated (*rho* = .336, *p* < .001).
